# Renal neuroendocrine tumors: clinical and molecular pathology with an emphasis on frequent association with ectopic Cushing syndrome

**DOI:** 10.1007/s00428-023-03596-5

**Published:** 2023-07-05

**Authors:** Atsuko Kasajima, Nicole Pfarr, Alexander von Werder, Kristina Schwamborn, Jürgen Gschwend, Nasir Ud Din, Irene Esposito, Wilko Weichert, Marianne Pavel, Abbas Agaimy, Günter Klöppel

**Affiliations:** 1 Department of Pathology, Technical University Munich, Trogerstr. 18, 81675 Munich, Germany; 2 Department of Internal Medicine II, Technical University Munich, Munich, Germany; 3 Department of Urology, Technical University Munich, Munich, Germany; 4 Section of Histopathology, Department of Pathology and Laboratory Medicine, Aga Khan University Hospital, Karachi, Pakistan; 5 Institute of Pathology, Heinrich-Heine University and University Hospital Düsseldorf, Düsseldorf, Germany; 6 Department of Internal Medicine, University Hospital Erlangen, Erlangen, Germany; 7 Department of Pathology, University Hospital Erlangen, Erlangen, Germany

**Keywords:** Neuroendocrine neoplasms, Kidney, ISL1, SATB2, Hormone

## Abstract

Renal neuroendocrine tumors (RenNETs) are rare malignancies with largely unknown biology, hormone expression, and genetic abnormalities. This study aims to improve our understanding of the RenNETs with emphasis of functional, hormonal, and genetic features. Surgically resected RenNETs (*N* = 13) were retrieved, and immunohistochemistry and next-generation sequencing (NGS) were performed in all cases. In addition, all published RenNETs were systematically reviewed. Our cohort (4 men and 9 women, mean age 42, mean tumor size 7.6 cm) included 2 patients with Cushing syndrome (CS). WHO grade (23% G1, 54% G2, and 23% G3) and tumor progression did not correlate. CS-associated RenNETs (CS-RenNETs) showed a solid and eosinophilic histology and stained for ACTH, while the remaining non-functioning tumors had a trabecular pattern and expressed variably hormones somatostatin (91%), pancreatic polypeptide (63%), glucagon (54%), and serotonin (18%). The transcription factors ISL1 and SATB2 were expressed in all non-functioning, but not in CS-RenNETs. NGS revealed no pathogenic alterations or gene fusions. In the literature review (*N* = 194), 15 (8%) of the patients had hormonal syndromes, in which CS being the most frequent (7/15). Large tumor size and presence of metastasis were associated with shorter patients’ survival (*p* < 0.01). RenNETs present as large tumors with metastases. CS-RenNETs differ through ACTH production and solid-eosinophilic histology from the non-functioning trabecular RenNETs that produce pancreas-related hormones and express ISL1 and SATB2. *MEN1* or *DAXX/ARTX* abnormalities and fusion genes are not detected in RenNETs, indicating a distinct yet unknown molecular pathogenesis.

**Supplementary Information:**

The online version contains supplementary material available at 10.1007/s00428-023-03596-5.

## Introduction

Well-differentiated neuroendocrine neoplasms (NETs) in genitourinary organs including renal NETs (RenNETs) are extremely rare compared to NETs occurring in digestive and thoracic organs [26]. About 200 RenNETs have been so far reported in the English literature, mostly as case reports or rare small series. RenNETs account for 0.18% (5/2780) of all primary renal neoplasms [37] and most arise in a normal kidney, but they may also occur in abnormal conditions, such as horseshoe kidney [20] and/or, rarely, within mature teratoma [30]. Regarding grading based on Ki67, no systematic evaluation of Ki67 has been performed to date in relation to patient outcome and metastatic potential in RenNETs [29].

The origin of RenNETs in the kidney is unclear since no neuroendocrine cells have so far been identified in normal renal tissue [57]. Apart from RenNETs, there are also poorly differentiated neuroendocrine neoplasms (carcinomas) in the kidney, most of them being small cell carcinomas and mixed non-neuroendocrine and endocrine neoplasms (MiNENs), mainly arising in the pelvis [38]. Few studies including altogether 16 RenNETs recorded the expression of hormones such as pancreatic polypeptide (PP), somatostatin, glucagon, or serotonin, but a systematic study on the hormonal profile in RenNETs is lacking [16, 22, 23, 32, 46, 53]. Transcription factors that are typical for the pancreas (islet 1, ISL1) [1], the lung (TTF1) [35], or the intestine (CDX2) [7] were examined in single case reports [12], while the expression of SATB2, a transcription factor characteristic for the lower digestive tract [13], has so far not been analyzed. Data on molecular genetic features of RenNETs are scarce and include one study based on NGS [44] and two studies focusing on loss of heterozygosity (LOH) on 3p [15, 34].

The discussion of the foregoing data shows that the available information on RenNETs is scarce. In particular, the prognostic assessment of RenNETs is poor compared to what is known in digestive tract NETs. Furthermore, it is unclear what biological relationship exists with pancreatic NETs (PanNET) and tumors with ACTH production. This study therefore focuses on four questions: (1) what is the prognostic significance of Ki67 index and WHO grade in RenNETs, (2) what is the incidence of functional syndromes in RenNETs, (3) are there histological and immunohistochemical features distinguishing non-functioning from Cushing syndrome associated RenNETs, and (4) are there molecular features that are unique to RenNETs?

## Materials and methods

### Tissue sampling and clinicopathological information

We identified surgically resected tumors diagnosed as primary renal neuroendocrine tumor or carcinoid over a period of 11 years (from 2011 to 2022) from the in-house surgical pathology files at the departments of pathology of the University Hospitals of Technical University Munich, University Hospital Düsseldorf, and University of Erlangen and also from the consultation files of two of the authors (AA and GK). NETs that had metastasized from other organs to the kidney were excluded in all cases by radiological and clinical findings. In all cases, formalin-fixed paraffin-embedded (FFPE) tissue blocks were available. The selected cases were reviewed and classified according to the WHO standards [48]. Nuclear Ki67 labeling was counted in more than 500 tumor cells in the area with the highest density (hot spot), and its percentage was reported as Ki67 index. Data on age, sex, tumor size and site, other renal diseases, hormonal symptoms, and presence or absence of metastasis were extracted from the available documents (see Table 1). This study was approved by the ethics committee of the Technical University Munich, Germany (approval number 2022–396-DFG-SR).

**Table 1:** Clinical features of patients with renal neuroendocrine tumor

ID	WHO	Functionality	Age	Sex	Size (cm)	Site	Microscopical	Ki67 index (%)	Islet 1	SATB2	Hormone	SST2	Metastasis at the time of diagnosis	Survival outcome DSS (months)
1	G1	NF	50	M	8.5	L	Trabecular	2	Diffuse	Diffuse	PP, SOM (patchy)	Score 2	None	24 months, alive/progress
2	G1	NF	39	F	12.5	L	Trabecular	2	Diffuse	Diffuse	SOM, GLU (diffuse) PP (patchy)	Score 0	n.d	n.d
3	G2	NF	42	M	7.0	L	Trabecular	3	Diffuse	Diffuse	SOM (patchy)	Score 1	Yes (lymph node)	60 months, alive/progress
4	G2	NF	27	F	7.5	R	Trabecular	4	Diffuse	Diffuse	SERO (diffuse) PP, GLU, SOM (patchy)	Score 2	Yes (lymph node)	58 months, alive/disease-free
5	G2	NF	50	F	5.0	R	Trabecular	5	Diffuse	Diffuse	SOM (patchy)	Score 1	Yes (liver, bone, soft tissue)	17 months, alive/progress
6	G2	NF	28	F	11.0	L	Trabecular	5	Diffuse	Diffuse	GLU, SOM, PP (patchy)	Score 0	Yes (lymph node)	n.d
7	G2	NF	43	F	8.8	L	Trabecular	7	Diffuse	Diffuse	GLU (diffuse)	Score 0	Yes (liver)	63 months, alive/progress
8	G2	NF	52	F	5.9	R	Trabecular	7	Diffuse	Diffuse	SOM (patchy)	Score 1	Yes (lymph node)	96 months, alive/progress
9	G2	NF	63	M	8.0	R	Trabecular	8	Diffuse	Diffuse	SOM (patchy)	Score 2	None	63 months, alive/progress
10	G3	NF	31	M	6.2	R	Trabecular	21	Diffuse	Diffuse	GLU (diffuse) PP (patchy)	Score 2	None	55 months, alive/progress^#^
11	G3	NF	42	F	8.0	R	Trabecular	23	Diffuse	Diffuse	GLU, SOM, SREO (patchy) PP (diffuse)	Score 3	Yes (lymph node)	12 months, alive/disease-free
12	G1	CS	32	F	4.5	R	Solid	2	Negative	Negative	ACTH (diffuse)	Score 1	None	48 months, alive/disease-free
13	G3	CS	54	F	3.6	R	Solid	33	Negative	Negative	ACTH (diffuse) SOM (patchy)	Score 0	none	57 months, alive/disease-free

*NET* neuroendocrine tumor, *NF* non-functioning, *CS* Cushing syndrome, *M* male, *F* female, *L* left, *R* right, *PP* pancreatic polypeptide, *SOM* somatostatin, *GLU* glucagon, *SERO* serotonin, *ACTH* adrenocorticotropes hormone, *SST2* somatostatin receptor 2, *DSS* disease specific survival, *n.d.* no data available

^#^Metachronous metastases appeared to the contralateral kidney and the pancreas after 24 and 48 months, respectively

### Histopathological and immunohistochemical evaluation

Immunohistochemical staining was performed on 2-µm sections using an automated system (Benchmark XT; Ventana/Roche, AZ, USA). Details regarding the immunohistochemical stainings are given in Supplementary Table 1. The expression was regarded as diffuse when all tumor cells were strongly and evenly stained or patchy when the staining of the tumor cells alternates between weak and strongly and the weakly stained cells dominated. A single cell positivity (< 5%) was regarded as negative. Membranous expression of the somatostatin receptor 2 (SST2) was evaluated based on the previously described method and a score 2 + and score 3 + were regarded as positive [42].

### Molecular genetic studies

DNA and RNA, respectively, were each extracted from 5 slides with 8-µm sections of FFPE materials that contain tumor tissue more than 50% of the section area. After proteinase k digestion, nucleic acids were extracted using a semi-automated extraction system (Maxwell RSC 48, Promega, Madison, USA). Nucleic acid quantity was fluorometrically measured using the DNA high-sensitivity kit or the RNA high-sensitivity kit and the QuBit 4.0 instrument (Thermo Fisher Scientific, Waltham, USA). The amount of amplifiable DNA (sequencing grade quality) was determined using a commercially available qPCR assay (TaqMan RNAse P detection assay) while RNA quality was determined by a custom-designed qPCR approach for amplification of the RNA of a housekeeping gene (HPRT) on a StepOnePlus instrument (Thermo Fisher Scientific). The DNA amount as input for library preparation was adjusted according to the amplifiability of the DNA and the grade of degradation. Hybrid capture and library preparation were conducted using the TruSight Oncology 500 assay (Illumina, San Diego, CA, USA), following the manufacturer’s protocol. This assay allows targeted-capture sequencing of 523 cancer-related genes on the DNA level and translocation detection of 50 driver fusion genes on the RNA level. Up to eight DNA/RNA pairs were pooled together and sequenced on a NextSeq 550DX (Illumina) system using a NextSeq 500/550 High Output Kit v2.5 (300 Cycles). Data was processed and analyzed by the TruSight Oncology 500 Local App version 2.11.3, followed by an in-house pipeline using a second variant caller (Mutect2) and ANNOVAR for annotation of the alterations [11, 54]. For DNA analysis, single nucleotide variants, insertions/deletions, copy number variations, total mutation burden (TMB), and microsatellite instability (MSI) were calculated. For RNA analysis, putative gene fusion of around 50 fusion driver genes and RNA splice variants from EGFR, AR, or MET (e.g., MET exon 14 skipping) were explored. TMB was calculated by dividing the total number of somatic single nucleotide variants and insertions/deletions by the length of the captured coding region (~ 1.24 Mb). MSI quantitative score was calculated by interrogating 130 homopolymer MSI marker sites and defined as the proportion of MSI unstable sites to the total MSI sites. Variants were checked for germline or somatic origin using the COSMIC (catalog of somatic mutations in cancer) database [17], dbSNP, and the gnomAD database [25]. Interpretation of variants was performed using OncoKb, Varsome, and CKB [9, 31, 43].

### Literature review and data collection

For a systematic review of RenNETs, we screened 204 articles written in English, Japanese, or German (14 articles in other languages reporting 18 cases were excluded) using the PubMed Keywords (renal[Title] AND (neuroendocrine tumor[Title])) OR (kidney[Title] AND (neuroendocrine tumor[Title])) OR (kidney[Title] AND (carcinoid[Title])) OR (renal[Title] AND (carcinoid[Title])). 61/204 articles dealing with non-renal NETs and 4 review articles were excluded. The remaining 139 articles included 122 case reports and 17 series-based articles with a total of 227 cases. Data on sex, age, site, location, other renal disease, hormonal syndromes, outcome, Ki67 index, WHO grade, and immunohistochemical and genetic features were extracted. Outcome data such as progression-free survival (PFS) were defined as duration between the initial diagnosis and the first tumor progression, tumor-related death, or last observation. Outcome data were available in 120 cases with a mean follow-up duration of 33 months.

### Statistical analyses

JMP Pro version 16.0.0 software (SAS Institute, Inc., Cary, NC, USA) was used for all statistical analyses. A correlation coefficient was calculated by Spearman’s method. For the comparison among clinicopathological data extracted from the previously published patients’ data, the sample number among multiple groups was compared using Pearson’s chi-squared test or Fischer’s exact test. The Wilcoxon test was applied for the comparisons of continuous values or scores between multiple groups found to be non-normally distributed by the Shapiro–Wilk test. The probability of differences in PFS was determined using the Kaplan–Meier method, with a log-rank test to test for significance. A *p* value of < 0.05 was considered statistically significant.

## Results

### Patient characteristics and clinical features

Thirteen RenNETs were identified (5 in-house, 8 consultations). Table 1 summarizes the most important clinical data. The mean age of the patients was 42 years (range 27–63). Metastasis was detected in 67% (8/12, data missing in 1 case) of the patients at the time of the first diagnosis. 62% (8/13) of the tumors were found in the right kidney, 38% in the left. None of the tumors was associated with horseshoe kidney. Two patients (17%) presented with Cushing syndrome; the other 11 patients (83%) had no hormone-related syndromes. No patients have multiple endocrine neoplasm type 1 (MEN1) or other hereditary/genetic tumor syndromes. The non-functioning tumors were significantly larger in size than the Cushing syndrome-associated tumors (mean 8 cm vs. 4 cm).

### WHO classification of RenNETs and its clinical correlation

Three NETs were graded as a G1 (23%), 7 as G2 (54%), and 3 as G3 (23%). The mean Ki67 index of the tumors was 9% (range 2 to 33). The WHO grade was not associated with sex, age, size, hormone-related symptoms, metastases, or patients’ outcome (Table 1).

### Macroscopic, histological, and immunohistochemical features

**Fig. 1:**
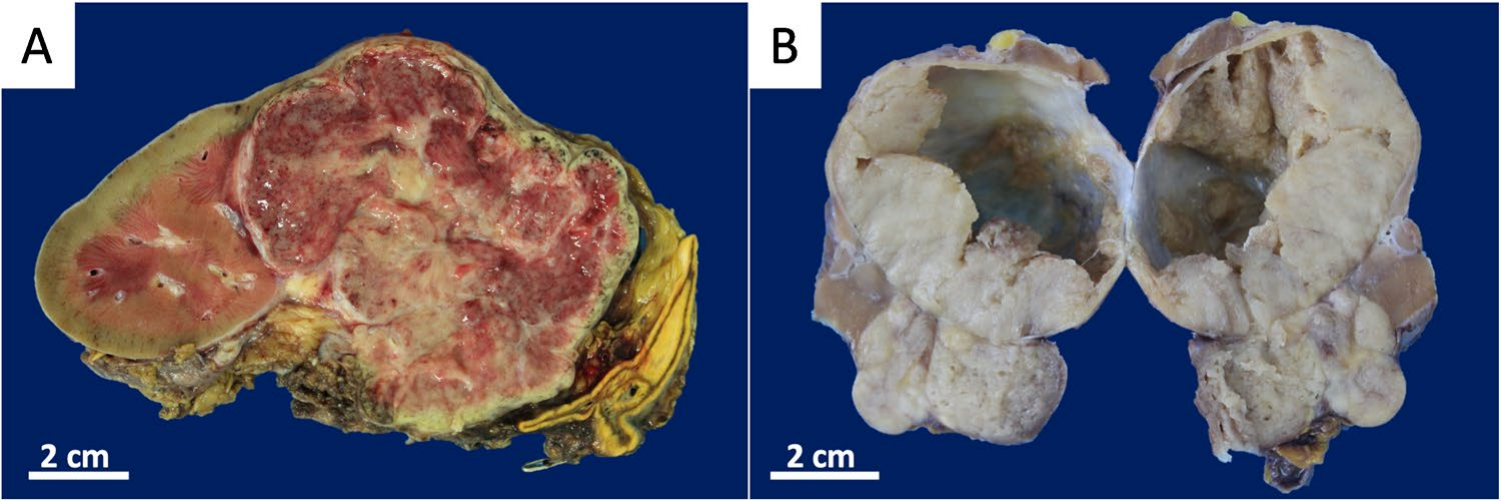
Gross findings of two renal neuroendocrine tumors. **A** A nephrectomy specimen with a 7.5 cm large tumor extending from the middle part to upper pole of the kidney. The tumor is well demarcated and partly lobulated in shape, showing a red-brown to yellow–brown cut surface with partly septal fibrosis. **B** A partial nephrectomy specimen with a 6.2 cm large multilobulated tumor. A part of the tumor shows a cystic change. The cut surface is gray-whitish and focally yellowish in color

**Fig. 2:**
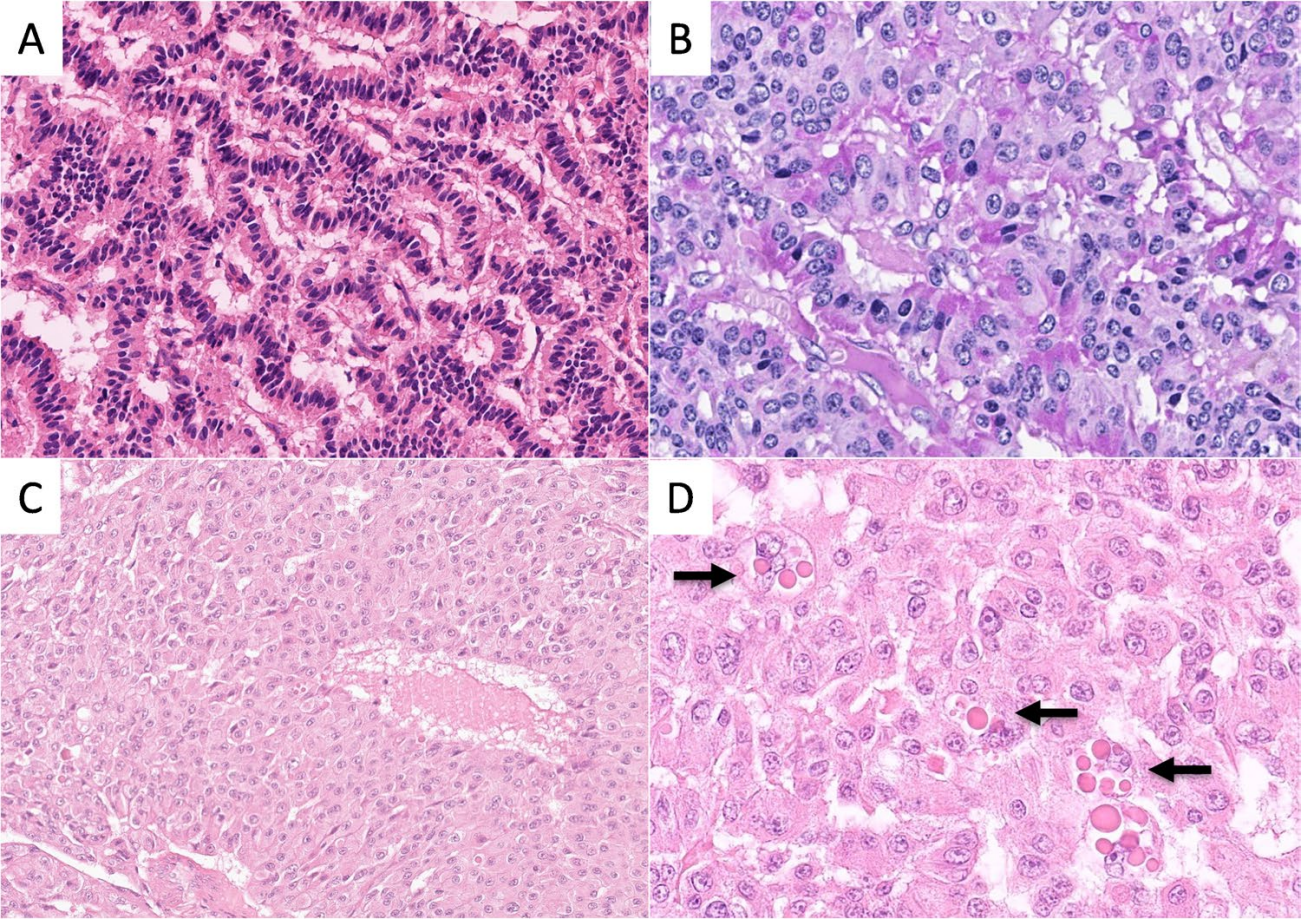
Histological features of a non-functioning renal neuroendocrine tumor (**A**, **B**) and a Cushing syndrome-associated renal neuroendocrine tumor (**C**, **D**). **A** Cylinder-shaped tumor cells arrange in a single layer trabecula that branch and anastomose each other (hematoxylin and eosin staining) **B** and focally cytoplasmic PAS (periodic acid-Schiff) positivity in a non-functioning tumor. **C** Polygonal tumor cells with a wide eosinophilic cytoplasm grow in solid nests in a Cushing syndrome-associated tumor. **D** Tumor cells showing prominent nucleoli and occasionally intracytoplasmic eosinophilic inclusions (arrows)

Grossly, 54% (7/13) of RenNETs were solid with a red-brown to yellow–brown cut surface (Fig. 1A) and the remaining tumors (46%) were partly cystic (Fig. 1B). Histologically, all non-functioning RenNETs had a distinct reticulated trabecular pattern (Fig. 2A, Table 1) with cubic cells slightly diastase-resistant PAS positive (Fig. 2B), while all the Cushing syndrome-associated RenNETs showed a solid growth pattern with relatively large eosinophilic and granular cells (Fig. 2C). The nuclei of these cells shaped irregular and displayed round macronucleolus and occasionally cytoplasmic eosinophilic inclusion bodies (Fig. 2D).

**Fig. 3:**
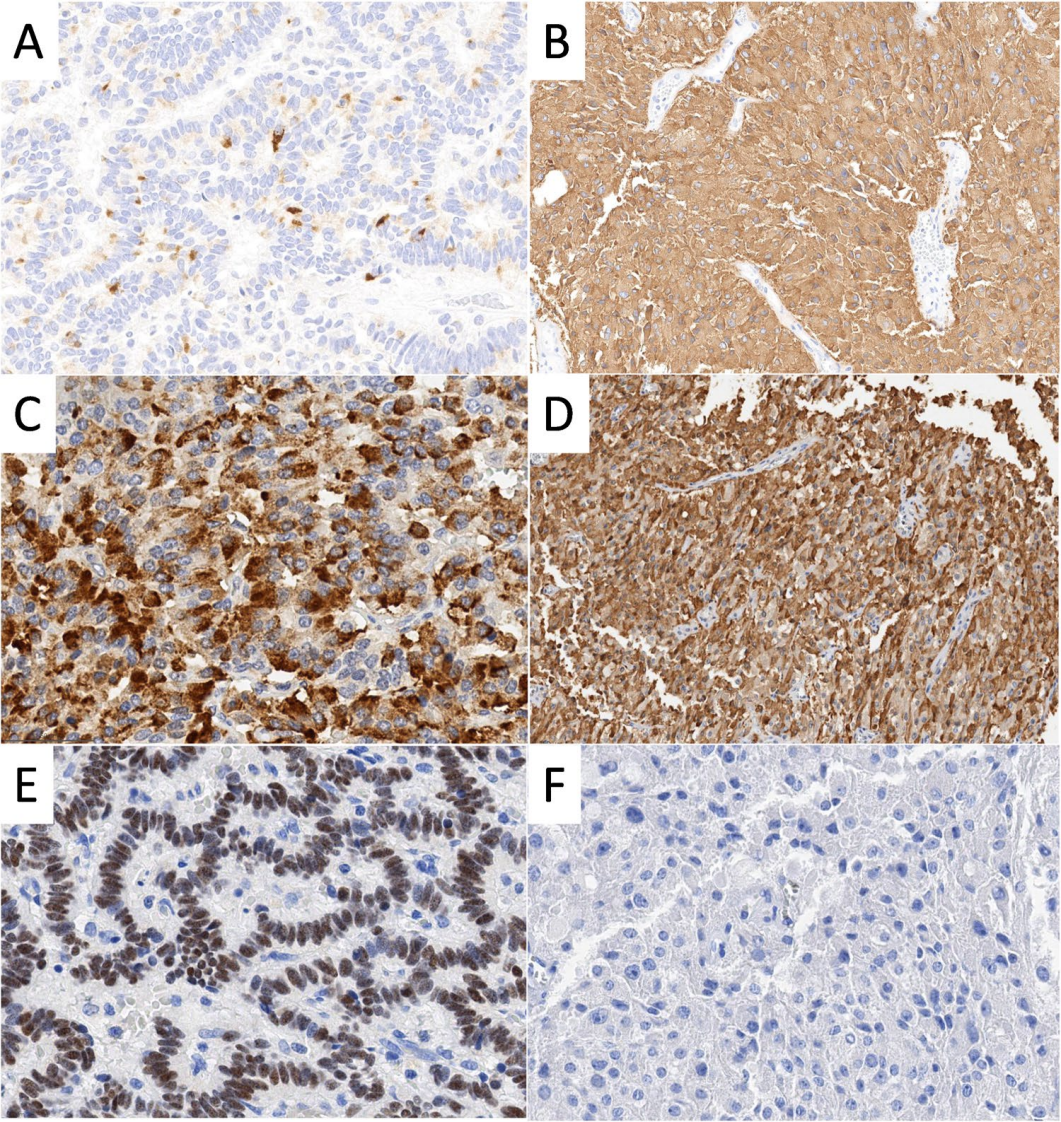
Immunohistochemical features of non-functioning (**A**, **C**, **E**) and Cushing syndrome-associated (**B**, **D**, **F**) renal neuroendocrine tumors. **A** Patchy expression of chromogranin A in a non-functioning renal NET and **B** diffuse and strong expression of chromogranin A in a Cushing-associated renal NET. **C** Diffuse somatostatin expression with heterogenous intensity in a non-functioning tumor. **D** Diffuse and strong ACTH expression in a Cushing syndrome-associated tumor. **E** Diffuse and strong nuclear expression of ISL1 in non-functioning tumor and **F** no expression of ISL1 in a Cushing syndrome-associated tumor

Immunohistochemically, all tumors were diffusely positive for CK18 and synaptophysin, while chromogranin A was diffusely expressed in both Cushing syndrome-associated RenNETs and in 3/11 non-functioning RenNETs (Fig. 3A, B). INSM1 was diffusely positive in all cases including those with a patchy chromogranin A staining. All non-functioning RenNETs expressed at least one of the pancreatic hormones (6 monohormonal, 5 multihormonal) with somatostatin in 91% (Fig. 3C), followed by PP in 63% and glucagon in 54% of the cases. Insulin and ACTH were negative in all non-functioning tumors. Both Cushing syndrome-associated RenNETs expressed diffusely ACTH (Fig. 3D), while all other hormones were negative except for a patchy somatostatin expression in one case. All non-functioning RenNETs expressed ISL1 (Fig. 3E), while Cushing-associated tumors were negative (Fig. 3F). SATB2 was diffusely positive in all non-functioning tumors and negative in Cushing syndrome cases. CDX2, TTF1, and PAX8 were consistently negative. Membranous SST2 labeling was found in 49% of non-functioning RenNETs but in none of the Cushing syndrome-associated RenNETs (Table 1).

### Genetic features

NGS was successfully performed in all tumors. The known NET-related gene alterations such as *ATRX*, *DAXX*, *MEN1*, or *TSC1/2* or NEC-related genes such as *TP53*, *RB1*, or *PIK3CA*-related genes were not detected in any of the cases. No gene with a pathogenic variant (class 5) was identified. Gene alterations with probably pathogenic variations (class 4) were found in three non-functioning tumors (23%) affecting *SDHA* (case 2), *ARID1B* (case 6), and *ASXL1* (case 9).

Gene alterations of class 3–T4 (variant of unclear significance with probably pathogenic tendency) were found in four non-functioning tumors affecting *PMS2* (case 11), *NUP93* (case 4), *TOP2A* (case 2), and *SNCAIP* (case 3). Other gene changes with unclear pathogenic significance (class 3) are listed in Supplementary Table 2. Fusion genes were not detected. The median value of TMB was 0.8 (range 0–5.5). The median MSI quantitative score was 1.72% (range 0–5.88). None of the cases showed a high MSI score (> 10). The tumor with the highest MSI score (5.88 in case 11) showed retained immunohistochemical staining for mismatch repair proteins (MLH1, PMS2, MSH2, MSH6). Loss of SDHB expression was not observed in two cases (case 9 and case 13) with class 2 *SDHD* mutations (Supplementary Table 2).

### Literature review

The clinical data of 227 RenNETs extracted from published articles is summarized in Table 2. Of the 194 cases in which hormonal status was documented, 15 (8%) had hormonal symptoms, of which Cushing syndrome was being the most frequent (7/15), followed by carcinoid syndrome (5/15). The remaining three RenNETs with hormonal syndrome included an insulin-secreting tumor with a hypoglycemic syndrome [45], a glucagon-producing tumor with a glucagonoma-like syndrome [18], and a tumor with a carcinoid-like syndrome [24]. Follow-up data were available in 120 cases (mean 33 months). Large tumor size (6 cm or larger, *p* = 0.01) and presence of metastasis at the time of diagnosis (*p* < 0.001) were significantly associated with poor patient outcome based on PFS, while age, sex, hormone-related syndrome, and horseshoe kidney were not. Data on WHO grade, available in only 58 cases, and Ki67 index, available in 41 cases, were not associated with outcome. Pancreatic hormone expression was reported to be positive in 60% (6/10) for glucagon, in 50% (7/14) for somatostatin, in 25% (3/12) for PP, and in 59% (7/12) for serotonin. The transcription factor ISL1 was examined in one case and was positive [12], while TTF1 and PAX8 were negative in all examined cases (30 and 27 cases, respectively). Molecular analysis was performed in four studies [15, 34, 44, 53]. Loss of heterozygosity (LOH) on 3p21 was reported in 5 of 11 cases [15, 34, 44]. NGS analysis was performed in 9 RenNETs and revealed variable mutation profiles [44]. The gene abnormalities which were most frequently found included mutations of *CDH1* and *TET2*, with three mutations in two cases. Next in frequency were LOH 3p and mutations in *AKT3*, *ROS1*, *PIK3P2*, *BCR*, and *MYC* [44].

**Table 2:** Clinical features of reported renal neuroendocrine tumors (1966 to 2023)

Clinical features		Number of available patients	Case number	%
		227		100
Sex	Female:male	218	125:93	57:43
Age	Median (range)	225	51 (10–87)	
Size (cm)	Median (range)	215	6.4 (1.2–25)	
Morphology	Solid:cystic	72	35:37	48:52
Site	Right:left*	173	101:72	58:42
WHO grade	G1:G2:G3	58	31:26:1	53:45:2
Ki67 index (%)	Mean (range)	41	4.8 (1–18)	
Location		111		
	Upper pole		23	14
	Middle, hilus		36	22
	Lower pole		44	27
	Entire		8	5
Other renal disease/condition		211		
	None		164	78
	Horseshoe kidney		36	16
	Combined tumor**		10	9
	Others***		3	0.1
Hormonal symptoms		194		
	None		166	86
	Cushing syndrome		7	4
	Carcinoid syndrome		5	3
	Insulinoma		1	1
	Glucagonoma-like		1	1
	Carcinoid syndrome-like		1	1
Metastasis		117		
	Absence		75	42
	Presence		102	58
Follow-up (months)	Mean (range)	120	33 (1–205)	
PFS rate				
	2 years		72%	
	5 years		44%	
	10 years		11%	

*PFS* progression-free survival

*21 cases (12%) in isthmus of horseshoe kidney

**8 cases combined with teratoma, 2 cases combined with cystadenoma

***Renal cell carcinoma in 2 cases, polycystic kidney in 1 case

## Discussion

Our study revealed that RenNETs constitute an entity of its own among the various NETs of the body. These usually large tumors have a size-dependent prognosis and a hormonal profile which is pancreas-like, including a high rate of ectopic Cushing syndrome.

All RenNETs in our study, the two Cushing syndrome cases excluded, presented as large tumors with a mean of 8 cm, setting them apart from most of the other NETs of the body such as the digestive (mean 2.6 cm for pancreas) [47] and pulmonary tract (mean 2.4 cm for the lung) [27] and larynx (mean 1.8 cm) [6] and making them comparable to thymus NETs that are usually also large with a similar mean size of 7 cm compared to RenNETs [50]. The reason for the remarkable large size of renal and thymic NETs is probably the location of both organs which allows a silent, symptomless growth for a long time. This unnoticed growth may also explain in RenNET the high rate of metastasis of 73% at diagnosis and in thymic NET the high rate of invasion into adjacent organs or metastasis, which is 60% [50]. Because our RenNET cohort is too small to allow any outcome evaluation, we therefore took the available data from our literature review and found that the 5-year PFS rate of RenNETs is 65% for tumors smaller than 6 cm compared to 31% for tumors 6 cm or larger.

Apart from size, our literature review also revealed that the presence of metastasis at the time of diagnosis has also a prognostic significance with a 5-year PFS rate of 26% vs. 84%in RenNETs without metastases. Surprisingly, we found that the WHO grade, as it is presented in Table 2, was not related to patients’ outcome. However, this finding has to be interpreted with caution since the extracted data from literature are limited and probably too small to allow yet any far-reaching conclusions. The reason is that the Ki67-based WHO grading system was only introduced in 2022 to the NETs of the urogenital organ systems [48]. Kim’s study from 2019 is the first which applied the Ki67 grading to RenNETs and showed in 6 cases that RenNETs with a Ki67 index above 3% are significantly more often associated with metastasis than those with a Ki67 of less than 3% [29]. Since the Ki67 values of RenNETs of our cohort are generally higher than those in Kim’s report, the Ki67 findings in our series of RenNETs, which did not correlate with presence of metastasis, are difficult to compare with those of Kim’s study. A study with a higher number of RenNET cases is therefore needed to clarify the prognostic role of the Ki67-based grading in this NET entity.

Two of our RenNETs presented with an ectopic Cushing syndrome. Hormonal syndromes were reported in 14 of 194 cases, of which data on functional activity were available. RenNETs with Cushing syndrome accounted for 50% of all syndromic cases or for 4% of all RenNETs. The second most common syndrome is the carcinoid syndrome, which has been reported in 36% of syndromic cases, but was absent in our cohort. Very rare are insulinoma and glucagonoma with one case each [18, 45]. The high frequency of 15% Cushing syndrome cases among our RenNETs reflects the selection bias that is always associated with a referral case series. However, even if the relative percentage of our Cushing RenNETs is too high, it indicates that an ectopic Cushing syndrome is a feature of this NET entity, which in terms of its frequency has not yet been properly appreciated. The relative frequency of 4% of a Cushing syndrome in RenNETs is comparable with its frequency in pulmonary NETs, in which an ectopic Cushing syndrome is thought to be most frequent in the body, accounting for 4.3% of all pulmonary NETs [36]. It thus seems that RenNETs belong to those NET entities that are most associated with an ectopic Cushing syndrome, which may apart from the pulmonary tumors also include pancreatic and thymic NETs [3, 14].

Regarding the prognosis of RenNETs associated with a Cushing syndrome, our literature search suggests that they share the same prognosis with the non-Cushing syndrome cases, although they present as small tumors with a mean size of 4 cm, probably because of the clinical symptoms that may lead to early detection of the tumors. The two Cushing tumors of our series were classified as G1 and G3, and both patients have no metastasis in the course. In the literature, the Cushing RenNETs had metastasis in half of the cases (3/6). Poorly differentiated NENs of the kidney, usually of the small cell type, have so far not been reported in association with an ectopic Cushing syndrome.

RenNETs with ACTH production and Cushing syndrome are distinct tumors because they not only produce ACTH but also exhibit a special histology. They have a solid histological pattern and an eosinophilic (oncocytic) cytology, which delineate these tumors from the non-functioning RenNETs, that are characterized by cuboidal cells forming a reticulated trabecular pattern. This solid-eosinophilic pattern was also found in the RenNETs with Cushing syndrome reported in the literature, in which exact histological descriptions and/or illustrations were available [10, 19].

ACTH expression in RenNETs was restricted to the patients with Cushing syndrome. ACTH was neither found in any of the trabecular tumors in our series nor in any other non-functioning RenNET reported in the literature. Since the number of our RenNETs which were screened for ACTH is small, it is possible that future studies in larger case series may find ACTH in non-functioning RenNETs, as it has been shown in pulmonary NETs that were systematically screened for ACTH [36].

Another finding that distinguishes the RenNETs with Cushing syndrome from the remaining RenNETs is the differential expression of transcription factors. ISL1 that plays a crucial role in embryogenesis and differentiation of pancreatic beta cells and is frequently expressed in PanNETs [1] but also in duodenal NETs (83%), rectal NETs (75%) [58], and middle ear NETs (100%) [2] was found to be expressed in all non-functioning RenNETs, but not in the Cushing syndrome cases. Similarly, SATB2 that labels the lower gastrointestinal epithelium, and the NETs of the rectum (81%) [58] and middle ear NETs (100%) [5], was only found in non-functioning RenNETs and not in Cushing syndrome-related RenNETs. In contrast, PAX8, TTF1, and CDX2, markers for renal carcinomas, pulmonary and thyroid neoplasms, and small intestine or the appendix, respectively, were constantly negative in all RenNETs of our series and in the cases of the literature [4, 21, 51, 55, 56].

The hormone production in non-functioning RenNETs has so far only been investigated in 14 cases, identifying either PP [23, 46, 53], serotonin [8, 28, 39, 41], or multiple hormones including somatostatin and glucagon [16, 32, 33, 46, 52]. In our series, all the non-functioning tumors expressed at least one of the pancreas hormones (excluding insulin) or serotonin. Although we had two tumors with serotonin expression, none of our RenNETs had a carcinoid syndrome that has been described in 5 of the previously reported cases [40].

Due to the characteristic trabecular morphology, ISL1, and pancreatic hormonal expression, we anticipated a possible genetic similarity between RenNETs and PanNETs. However, none of the investigated tumors showed *MEN1*, *ATRX*/*DAXX* gene alterations that are detected in approx. 40% of PanNETs [49]. Instead, variable genes were affected in single cases without a definitive pathogenicity. Our results, together with a previous study, indicate that the tumorigenesis of RenNETs is, despite histological and immunohistochemical commonalities with PanNETs, distinct from that of PanNETs. This molecular distinction also argues against an origin of RenNETs from heterotopic pancreatic tissue in the kidney. Moreover, we were unable to find any report on heterotopic pancreatic tissue in the kidney.

In conclusion, RenNETs represent a small but distinct group of NETs. They manifest usually as large tumors with a size above 6 cm, a size that is of prognostic significance. Most RenNETs have a characteristic reticulated trabecular morphology, consistently coexpress ISL1 and SATB2, and are non-functioning, although they express a variety of entero-pancreatic hormones. Biologically and structurally distinct from these RenNETs are the ACTH-positive RenNETs associated with an ectopic Cushing syndrome and displaying a typical solid-eosinophilic morphology in the absence of ISL1 or SATB2 expression. Our literature review reveals that these ACTH-positive tumors belong to the group of NETs that are most frequently associated with an ectopic Cushing syndrome, such as bronchial, pancreatic, and thymic NETs. The genomic profile completely distinguishes RenNETs from pancreatic NETs including those with a Cushing syndrome.

### Supplementary Information

Below is the link to the electronic supplementary material.

Supplementary file1 (XLSX 18 KB)

## References

[1] Agaimy A, Erlenbach-Wunsch K, Konukiewitz B, Schmitt AM, Rieker RJ, Vieth M, Kiesewetter F, Hartmann A, Zamboni G, Perren A, Klöppel G (2013). ISL1 expression is not restricted to pancreatic well-differentiated neuroendocrine neoplasms, but is also commonly found in well and poorly differentiated neuroendocrine neoplasms of extrapancreatic origin. Mod Pathol.

[2] Agaimy A, Lell M, Schaller T, Markl B, Hornung J (2015). ‘Neuroendocrine’ middle ear adenomas: consistent expression of the transcription factor ISL1 further supports their neuroendocrine derivation. Histopathology.

[3] Agaimy A, Kasajima A, Stoehr R, Haller F, Schubart C, Togel L, Pfarr N, von Werder A, Pavel ME, Sessa F, Uccella S, La Rosa S, Kloppel G (2023). Gene fusions are frequent in ACTH-secreting neuroendocrine neoplasms of the pancreas, but not in their non-pancreatic counterparts. Virchows Arch.

[4] Amin M, Trikalinos N, Chatterjee D (2021). Single institutional experience on primary neuroendocrine neoplasms of the kidney: a rare distinct entity. Hum Pathol.

[5] Asa SL, Arkun K, Tischler AS, Qamar A, Deng FM, Perez-Ordonez B, Weinreb I, Bishop JA, Wenig BM, Mete O (2021) Middle ear “adenoma”: a neuroendocrine tumor with predominant L cell differentiation. Endocr Pathol. 10.1007/s12022-021-09684-z 10.1007/s12022-021-09684-z 34041698

[6] Bal M, Sharma A, Rane SU, Mittal N, Chaukar D, Prabhash K, Patil A (2022). Neuroendocrine neoplasms of the larynx: a clinicopathologic analysis of 27 neuroendocrine tumors and neuroendocrine carcinomas head neck. Pathology.

[7] Barbareschi M, Roldo C, Zamboni G, Capelli P, Cavazza A, Macri E, Cangi MG, Chilosi M, Doglioni C (2004). CDX-2 homeobox gene product expression in neuroendocrine tumors: its role as a marker of intestinal neuroendocrine tumors. Am J Surg Pathol.

[8] Begin LR, Guy L, Jacobson SA, Aprikian AG (1998). Renal carcinoid and horseshoe kidney: a frequent association of two rare entities–a case report and review of the literature. J Surg Oncol.

[9] Chakravarty D, Gao J, Phillips SM, Kundra R, Zhang H, Wang J, Rudolph JE, Yaeger R, Soumerai T, Nissan MH, Chang MT, Chandarlapaty S, Traina TA, Paik PK, Ho AL, Hantash FM, Grupe A, Baxi SS, Callahan MK, Snyder A, Chi P, Danila D, Gounder M, Harding JJ, Hellmann MD, Iyer G, Janjigian Y, Kaley T, Levine DA, Lowery M, Omuro A, Postow MA, Rathkopf D, Shoushtari AN, Shukla N, Voss M, Paraiso E, Zehir A, Berger MF, Taylor BS, Saltz LB, Riely GJ, Ladanyi M, Hyman DM, Baselga J, Sabbatini P, Solit DB, Schultz N (2017) OncoKB: a precision oncology knowledge base. JCO Precis Oncol 2017. 10.1200/PO.17.00011 10.1200/PO.17.00011 PMC5586540 28890946

[10] Chunharojrith P, Pradniwat K, Kongmalai T (2021) A rare case of ectopic ACTH syndrome caused by primary renal neuroendocrine tumor. Endocrinol Diabetes Metab Case Reports 2021. 10.1530/EDM-20-0076 10.1530/EDM-20-0076 PMC8052562 33847280

[11] Cibulskis K, Lawrence MS, Carter SL, Sivachenko A, Jaffe D, Sougnez C, Gabriel S, Meyerson M, Lander ES, Getz G (2013). Sensitive detection of somatic point mutations in impure and heterogeneous cancer samples. Nat Biotechnol.

[12] Deacon MJ, Harvey H, Shah C, Khan A (2021). A rare case of a large primary renal neuroendocrine tumour: a case report and brief review of literature. Cureus.

[13] Dum D, Kromm D, Lennartz M, De Wispelaere N, Buscheck F, Luebke AM, Burandt E, Menz A, Kluth M, Hube-Magg C, Hinsch A, Hoflmayer D, Weidemann S, Fraune C, Moller K, Lebok P, Sauter G, Simon R, Uhlig R, Wilczak W, Minner S, Krech R, Bernreuther C, Marx A, Steurer S, Jacobsen F, Clauditz T, Krech T (2022). SATB2 expression in human tumors. Arch Pathol Lab Med.

[14] Ejaz S, Vassilopoulou-Sellin R, Busaidy NL, Hu MI, Waguespack SG, Jimenez C, Ying AK, Cabanillas M, Abbara M, Habra MA (2011). Cushing syndrome secondary to ectopic adrenocorticotropic hormone secretion: the University of Texas MD Anderson Cancer Center Experience. Cancer.

[15] el-Naggar AK, Troncoso P, Ordonez NG (1995). Primary renal carcinoid tumor with molecular abnormality characteristic of conventional renal cell neoplasms. Diagn Mol Pathol Am J Surg Pathol B.

[16] Fetissof F, Benatre A, Dubois MP, Lanson Y, Arbeille-Brassart B, Jobard P (1984). Carcinoid tumor occurring in a teratoid malformation of the kidney. An immunohistochemical study. Cancer.

[17] Forbes SA, Beare D, Gunasekaran P, Leung K, Bindal N, Boutselakis H, Ding M, Bamford S, Cole C, Ward S, Kok CY, Jia M, De T, Teague JW, Stratton MR, McDermott U, Campbell PJ (2015). COSMIC: exploring the world’s knowledge of somatic mutations in human cancer. Nucleic Acids Res.

[18] Gleeson MH, Bloom SR, Polak JM, Henry K, Dowling RH (1971). Endocrine tumour in kidney affecting small bowel structure, motility, and absorptive function. Gut.

[19] Hannah J, Lippe B, Lai-Goldman M, Bhuta S (1988). Oncocytic carcinoid of the kidney associated with periodic Cushing’s syndrome. Cancer.

[20] Hansel DE, Epstein JI, Berbescu E, Fine SW, Young RH, Cheville JC (2007). Renal carcinoid tumor: a clinicopathologic study of 21 cases. Am J Surg Pathol.

[21] Hartman MS, Mittal P, Lewis M (2006). Multifocal renal carcinoid tumor arising in horseshoe kidney with metastases to the thyroid. Radiol Case Rep.

[22] Huettner PC, Bird DJ, Chang YC, Seiler MW (1991). Carcinoid tumor of the kidney with morphologic and immunohistochemical profile of a hindgut endocrine tumor: report of a case. Ultrastruct Pathol.

[23] Isobe H, Takashima H, Higashi N, Murakami Y, Fujita K, Hanazawa K, Fujime M, Matsumoto T (2000). Primary carcinoid tumor in a horseshoe kidney. Int J Urol.

[24] Jhang S, Chiu AW (2021). An infertile female delivered a baby after removal of primary renal carcinoid tumor. Open Med (Wars).

[25] Karczewski KJ, Francioli LC, Tiao G, Cummings BB, Alfoldi J, Wang Q, Collins RL, Laricchia KM, Ganna A, Birnbaum DP, Gauthier LD, Brand H, Solomonson M, Watts NA, Rhodes D, Singer-Berk M, England EM, Seaby EG, Kosmicki JA, Walters RK, Tashman K, Farjoun Y, Banks E, Poterba T, Wang A, Seed C, Whiffin N, Chong JX, Samocha KE, Pierce-Hoffman E, Zappala Z, O'Donnell-Luria AH, Minikel EV, Weisburd B, Lek M, Ware JS, Vittal C, Armean IM, Bergelson L, Cibulskis K, Connolly KM, Covarrubias M, Donnelly S, Ferriera S, Gabriel S, Gentry J, Gupta N, Jeandet T, Kaplan D, Llanwarne C, Munshi R, Novod S, Petrillo N, Roazen D, Ruano-Rubio V, Saltzman A, Schleicher M, Soto J, Tibbetts K, Tolonen C, Wade G, Talkowski ME, Neale BM, Daly MJ, MacArthur DG, Genome Aggregation Database C (2020). The mutational constraint spectrum quantified from variation in 141,456 humans. Nature.

[26] Kasajima A, Klöppel G (2020). Neuroendocrine neoplasms of lung, pancreas and gut: a morphology-based comparison. Endocr Relat Cancer.

[27] Kasajima A, Ishikawa Y, Iwata A, Steiger K, Oka N, Ishida H, Sakurada A, Suzuki H, Kameya T, Konukiewitz B, Kloppel G, Okada Y, Sasano H, Weichert W (2018). Inflammation and PD-L1 expression in pulmonary neuroendocrine tumors. Endocr Relat Cancer.

[28] Kawahara T, Nagashima Y, Misaki H (2009). Primary renal carcinoid tumor with a mucinous cystadenoma element. Int J Urol.

[29] Kim B, Kim HS, Moon KC (2019). Primary renal well-differentiated neuroendocrine tumors: report of six cases with an emphasis on the Ki-67 index and mitosis. Diagn Pathol.

[30] Kojiro M, Ohishi H, Isobe H (1976). Carcinoid tumor occurring in cystic teratoma of the kidney: a case report. Cancer.

[31] Kopanos C, Tsiolkas V, Kouris A, Chapple CE, Albarca Aguilera M, Meyer R, Massouras A (2019). VarSome: the human genomic variant search engine. Bioinformatics.

[32] Kurl S, Rytkonen H, Farin P, Ala-Opas M, Soimakallio S (1996). A primary carcinoid tumor of the kidney: a case report and review of the literature. Abdom Imaging.

[33] Kuroda N, Katto K, Tamura M, Shiotsu T, Hes O, Michal M, Nagashima Y, Ohara M, Hirouchi T, Mizuno K, Hayashi Y, Lee GH (2008). Carcinoid tumor of the renal pelvis: consideration on the histogenesis. Pathol Int.

[34] Kuroda N, Alvarado-Cabrero I, Sima R, Hes O, Michal M, Kinoshita H, Matsuda T, Ohe C, Sakaida N, Uemura Y, Lee GH (2010). Renal carcinoid tumor: an immunohistochemical and molecular genetic study of four cases. Oncol Lett.

[35] La Rosa S, Chiaravalli AM, Placidi C, Papanikolaou N, Cerati M, Capella C (2010). TTF1 expression in normal lung neuroendocrine cells and related tumors: immunohistochemical study comparing two different monoclonal antibodies. Virchows Arch.

[36] La Rosa S, Volante M, Uccella S, Maragliano R, Rapa I, Rotolo N, Inzani F, Siciliani A, Granone P, Rindi G, Dominioni L, Capella C, Papotti M, Sessa F, Imperatori A (2019). ACTH-producing tumorlets and carcinoids of the lung: clinico-pathologic study of 63 cases and review of the literature. Virchows Arch.

[37] Lane BR, Chery F, Jour G, Sercia L, Magi-Galluzzi C, Novick AC, Zhou M (2007). Renal neuroendocrine tumours: a clinicopathological study. BJU Int.

[38] Lee SY, Hsu HH, Lin HY, Chen YC, Wong YC, Wang LJ, Ng KF, Chuang CK, Hung CC, Yang CW (2013). Factors associated with the survival of patients with primary small cell carcinoma of the kidney. Int J Clin Oncol.

[39] Lodding P, Hugosson J, Hansson G (1997). Primary carcinoid tumour with ossification masquerading as calyx stone in a horseshoe kidney. Scand J Urol Nephrol.

[40] McGarrah PW, Westin GFM, Hobday TJ, Scales JA, Ingimarsson JP, Leibovich BC, Halfdanarson TR (2020). Renal neuroendocrine neoplasms: a single-center experience. Clin Genitourin Cancer.

[41] Molinie V, Liguory Brunaud MD, Chiche R (1992). Primary carcinoid tumor of the kidney. Apropos of a case with immunohistochemical study. Archives d'anatomie et de cytologie pathologiques.

[42] Oka N, Kasajima A, Konukiewitz B, Sakurada A, Okada Y, Kameya T, Weichert W, Ishikawa Y, Suzuki H, Sasano H, Klöppel G (2020). Classification and prognostic stratification of bronchopulmonary neuroendocrine neoplasms. Neuroendocrinology.

[43] Patterson SE, Liu R, Statz CM, Durkin D, Lakshminarayana A, Mockus SM (2016). The clinical trial landscape in oncology and connectivity of somatic mutational profiles to targeted therapies. Hum Genomics.

[44] Pivovarcikova K, Agaimy A, Martinek P, Alaghehbandan R, Perez-Montiel D, Alvarado-Cabrero I, Rogala J, Kuroda N, Rychly B, Gasparov S, Michalova K, Michal M, Hora M, Pitra T, Tuckova I, Laciok S, Mareckova J, Hes O (2019). Primary renal well-differentiated neuroendocrine tumour (carcinoid): next-generation sequencing study of 11 cases. Histopathology.

[45] Ramkumar S, Dhingra A, Jyotsna V, Ganie MA, Das CJ, Seth A, Sharma MC, Bal CS (2014). Ectopic insulin secreting neuroendocrine tumor of kidney with recurrent hypoglycemia: a diagnostic dilemma. BMC Endocr Disord.

[46] Raslan WF, Ro JY, Ordonez NG, Amin MB, Troncoso P, Sella A, Ayala AG (1993). Primary carcinoid of the kidney. Immunohistochemical and ultrastructural studies of five patients. Cancer.

[47] Rindi G, Klersy C, Albarello L, Baudin E, Bianchi A, Buchler MW, Caplin M, Couvelard A, Cros J, de Herder WW, Delle Fave G, Doglioni C, Federspiel B, Fischer L, Fusai G, Gavazzi F, Hansen CP, Inzani F, Jann H, Komminoth P, Knigge UP, Landoni L, La Rosa S, Lawlor RT, Luong TV, Marinoni I, Panzuto F, Pape UF, Partelli S, Perren A, Rinzivillo M, Rubini C, Ruszniewski P, Scarpa A, Schmitt A, Schinzari G, Scoazec JY, Sessa F, Solcia E, Spaggiari P, Toumpanakis C, Vanoli A, Wiedenmann B, Zamboni G, Zandee WT, Zerbi A, Falconi M (2018). Competitive testing of the WHO 2010 versus the WHO 2017 grading of pancreatic neuroendocrine neoplasms: data from a large international cohort study. Neuroendocrinology.

[48] Rindi G, Moch H, McCluggage WG, Travis WD, Osamura RY, Papotti M, de Herder W (2022) Neuroendocrine neoplasms, non-endocrine organs. In: Board. WCoTE (ed) WHO Classification of Tumours. Endocrine and Neuroendocrine Tumours, 5th.edn. International Agency for Research on Cancer (IARC), Lyon, France, pp

[49] Scarpa A, Chang DK, Nones K, Corbo V, Patch AM, Bailey P, Lawlor RT, Johns AL, Miller DK, Mafficini A, Rusev B, Scardoni M, Antonello D, Barbi S, Sikora KO, Cingarlini S, Vicentini C, McKay S, Quinn MC, Bruxner TJ, Christ AN, Harliwong I, Idrisoglu S, McLean S, Nourse C, Nourbakhsh E, Wilson PJ, Anderson MJ, Fink JL, Newell F, Waddell N, Holmes O, Kazakoff SH, Leonard C, Wood S, Xu Q, Nagaraj SH, Amato E, Dalai I, Bersani S, Cataldo I, Dei Tos AP, Capelli P, Davi MV, Landoni L, Malpaga A, Miotto M, Whitehall VL, Leggett BA, Harris JL, Harris J, Jones MD, Humphris J, Chantrill LA, Chin V, Nagrial AM, Pajic M, Scarlett CJ, Pinho A, Rooman I, Toon C, Wu J, Pinese M, Cowley M, Barbour A, Mawson A, Humphrey ES, Colvin EK, Chou A, Lovell JA, Jamieson NB, Duthie F, Gingras MC, Fisher WE, Dagg RA, Lau LM, Lee M, Pickett HA, Reddel RR, Samra JS, Kench JG, Merrett ND, Epari K, Nguyen NQ, Zeps N, Falconi M, Simbolo M, Butturini G, Van Buren G, Partelli S, Fassan M, Khanna KK, Gill AJ, Wheeler DA, Gibbs RA, Musgrove EA, Bassi C, Tortora G, Pederzoli P, Pearson JV, Waddell N, Biankin AV, Grimmond SM, Australian Pancreatic Cancer Genome I (2017). Whole-genome landscape of pancreatic neuroendocrine tumours. Nature.

[50] Sullivan JL, Weksler B (2017). Neuroendocrine tumors of the thymus: analysis of factors affecting survival in 254 patients. Ann Thorac Surg.

[51] Sun K, You Q, Zhao M, Yao H, Xiang H, Wang L (2013). Concurrent primary carcinoid tumor arising within mature teratoma and clear cell renal cell carcinoma in the horseshoe kidney: report of a rare case and review of the literature. Int J Clin Exp Pathol.

[52] Takashi M, Matsuyama M, Furuhashi K, Kodama Y, Shinzato M, Shamoto M, Nakashima N (2003). Composite tumor of mucinous cystadenoma and somatostatinoma of the kidney. Int J Urol.

[53] van den Berg E, Gouw AS, Oosterhuis JW, Storkel S, Dijkhuizen T, Mensink HJ, de Jong B (1995). Carcinoid in a horseshoe kidney. Morphol Immunohistochem Cytogenet Cancer Genet Cytogenet.

[54] Wang K, Li M, Hakonarson H (2010). ANNOVAR: functional annotation of genetic variants from high-throughput sequencing data. Nucleic Acids Res.

[55] Wang XH, Lu X, He B, Jiang YX, Yu WJ, Wang H, Zhang W, Li YJ (2018). Clinicopathologic features of primary renal neuroendocrine carcinoma. Zhonghua Bing Li Xue Za Zhi.

[56] Zekri J, Rasool HJ, Meliti A, Rasool J (2019). Neuroendocrine tumor of the kidney: diagnostic challenge and successful therapy. Urol Ann.

[57] Zhang Q, Ming J, Zhang S, Qiu X (2012). Primary micro neuroendocrine tumor arising in a horseshoe kidney with cyst: report of a case and review of literature. Diagn Pathol.

[58] Zhao LH, Chen C, Mao CY, Xiao H, Fu P, Xiao HL, Wang G (2019). Value of SATB2, ISL1, and TTF1 to differentiate rectal from other gastrointestinal and lung well-differentiated neuroendocrine tumors. Pathol Res Pract.

